# The Use of Behavior Change Theory in Internet-Based Asthma Self-Management Interventions: A Systematic Review

**DOI:** 10.2196/jmir.4110

**Published:** 2015-04-02

**Authors:** Mustafa Al-Durra, Monika-Bianca Torio, Joseph A Cafazzo

**Affiliations:** ^1^Centre for Global eHealth Innovation, Techna Institute, University Health NetworkToronto, ONCanada; ^2^Institute of Health Policy, Management and EvaluationThe Dalla Lana School of Public HealthUniversity of TorontoToronto, ONCanada; ^3^Faculty of Applied Science & EngineeringUniversity of TorontoToronto, ONCanada; ^4^Institute of Biomaterials and Biomedical EngineeringUniversity of TorontoToronto, ONCanada

**Keywords:** asthma, self-care, self-management, eHealth, mHealth, mobile health, telehealth, telemedicine, Internet, review

## Abstract

**Background:**

The high prevalence rate of asthma represents a major societal burden. Advancements in information technology continue to affect the delivery of patient care in all areas of medicine. Internet-based solutions, social media, and mobile technology could address some of the problems associated with increasing asthma prevalence.

**Objective:**

This review evaluates Internet-based asthma interventions that were published between 2004 and October 2014 with respect to the use of behavioral change theoretical frameworks, applied clinical guidelines, and assessment tools.

**Methods:**

The search term (Asthma AND [Online or Internet or Mobile or Application or eHealth or App]) was applied to six bibliographic databases (Ovid MEDLINE, PubMed, BioMed Central, ProQuest Computing, Web of Knowledge, and ACM Digital Library) including only English-language articles published between 2004 and October 2014. In total, 3932 articles matched the priori search terms and were reviewed by the primary reviewer based on their titles, index terms, and abstracts. The matching articles were then screened by the primary reviewer for inclusion or exclusion based on their abstract, study type, and intervention objectives with respect to the full set of priori inclusion and exclusion criteria; 331 duplicates were identified and removed. A total of 85 articles were included for in-depth review and the remaining 3516 articles were excluded. The primary and secondary reviewer independently reviewed the complete content of the 85 included articles to identify the applied behavioral change theories, clinical guidelines, and assessment tools. Findings and any disagreement between reviewers were resolved by in-depth discussion and through a consolidation process for each of the included articles.

**Results:**

The reviewers identified 17 out of 85 interventions (20%) where at least one model, framework, and/or construct of a behavioral change theory were applied. The review identified six clinical guidelines that were applied across 30 of the 85 interventions (35%) as well as a total of 21 assessment tools that were applied across 32 of the 85 interventions (38%).

**Conclusions:**

The findings of this literature review indicate that the majority of published Internet-based interventions do not use any documented behavioral change theory, clinical guidelines, and/or assessment tools to inform their design. Further, it was found that the application of clinical guidelines and assessment tools were more salient across the reviewed interventions. A consequence, as such, is that many Internet-based asthma interventions are designed in an ad hoc manner, without the use of any notable evidence-based theoretical frameworks, clinical guidelines, and/or assessment tools.

##  Introduction

Asthma is a common chronic inflammatory disease of the airways with symptoms including cough, breathlessness, and wheezing. According to World Health Organization (WHO) estimates, there are some 235 million people in the world currently suffering from asthma. The WHO also estimates that asthma is the most common non-communicable disease among children [1,2].

Combined with the aging population trend and increasing cost of health care services, the high prevalence rate of asthma represents a major societal burden as well as a substantial challenge to the traditional models of health care providers, patients, and their families. A number of cost analysis studies have reported that the annual economic cost of asthma due to direct medical costs from hospital stays, as well as indirect costs from lost school and workdays, amounted to more than US $56 billion in the United States in 2007, CAN $1.8 billion in Ontario, Canada in 2011, and €19.3 billion in European adult populations in 2010 [3-5].

Advancements in the field of information technology continue to change patient care in all areas of medicine. Internet-based solutions, social media, and mobile technology could help to mitigate some of the problems associated with the increasing asthma prevalence [6].

The National Heart, Lung, and Blood Institute Expert Panel Report 3 (NHLBI EP3) Asthma Guidelines suggest that there is a potential use for information technologies to provide patients with skills to control their asthma and improve outcomes [7,8].

None of the existing literature reviews focused on evaluating Internet-based asthma interventions with respect to the evidence base around the behavioral change theoretical frameworks, applied clinical guidelines, and assessment tools.

The primary objective of this literature review was to identify and evaluate Internet-based asthma interventions that were published between 2004 and October 2014 with respect to the use of the behavioral change, self-care, and self-management theoretical frameworks as well as the application of clinical guidelines and assessment tools.

##  Methods

### Research Questions

We established the following primary research question: What is the use of behavioral change, self-care, and self-management theoretical frameworks within the context of Internet-based asthma interventions?

Our secondary research question was: What is the use of clinical guidelines and assessment tools within the context of Internet-based asthma interventions?

### Inclusion Criteria

The review included all asthma-related Internet-based interventions, such as Internet-based applications, electronic diary solutions, mobile apps, and/or any other kind of computer-based applications with the focus on patient-centric Internet-based applications as well as provider-to-patient applications.

The bibliographic databases search included relevant studies and interventions that were published between 2004 and October 2014 and was limited to literature published in the English language**.**

### Exclusion Criteria

The review excluded any electronic record management systems that are provider-centric and used to organize patient visits at the clinic and/or hospital settings such as electronic medical records (EMR), electronic health records (EHR), and hospital information systems (HIS). Also, telemedicine interventions that merely leveraged the conventional wired or wireless telephone technology as a medium to facilitate a verbal communication and/or short message service (SMS) between patients and their providers were excluded. The review excluded any educational-only studies that utilized Web-based resources, such as social media, decision support tools, and wikis, for the sole purpose of providing educational content for asthma patients, caregivers, and/or providers.

The bibliographic databases search excluded studies whose main objective was to design, develop, and assess eHealth tools, such as Web, Internet, and mobile apps, without providing critical analysis of their impact and contribution within a given asthma intervention context.

### Search Strategy

#### Overview

The search term was applied to six bibliographic databases (Ovid MEDLINE, PubMed, BioMed Central, ProQuest Computing, Web of Knowledge, and ACM Digital Library) including only English articles published between 2004 and October 2014. The search was conducted in the following steps.

#### Search Term

We limited the search to English-language articles published between 2004 and October 2014. The search term was:

- [Asthma] AND
- [English language and year="2004 -Current"] AND
- [Online or Internet or Mobile or Application or eHealth or App]

#### Step I — Abstract Evaluation

The primary reviewer evaluated the abstracts, titles, and index terms of all matching articles in the bibliographic databases where the search term was applied. Based on this preliminary review, all relevant articles were listed for potential inclusion.

#### Step II — Screening for Inclusion

In this step, the primary reviewer evaluated relevant articles in the preliminary list for final inclusion or exclusion based on their abstract, study type, and intervention focus with respect to the full set of priori inclusion and exclusion criteria.

#### Step III — Removal of Duplicates

Internal and cross-database duplicates were identified and removed from all included articles from Step II. Duplicates within each database were first identified and removed. Cross-database duplicates were then identified and removed through a manual consolidation process.

#### Step IV — Independent Review

The complete published papers of all included articles were then reviewed, analyzed, and assessed thoroughly by two reviewers independently. The primary and secondary reviewers independently reviewed the complete content of the included articles to identify the applied theoretical frameworks, clinical guidelines, and assessment tools with the objective to answer the priori research questions. Findings and disagreements between the primary and secondary reviewers were resolved by in-depth discussions and through a consolidation process for each of the included articles.

## Results

### Overview

In total, and across all six bibliographic databases, 3932 articles matched the priori search terms and were reviewed by the primary reviewer based on their titles, index terms, and abstracts in Step I.

In Step II, 3516 articles were excluded by the primary reviewer on their abstract, study type, and intervention focus that met the priori exclusion criteria.

A total of 331 duplicates were identified and removed in Step III.

**Figure 1:**
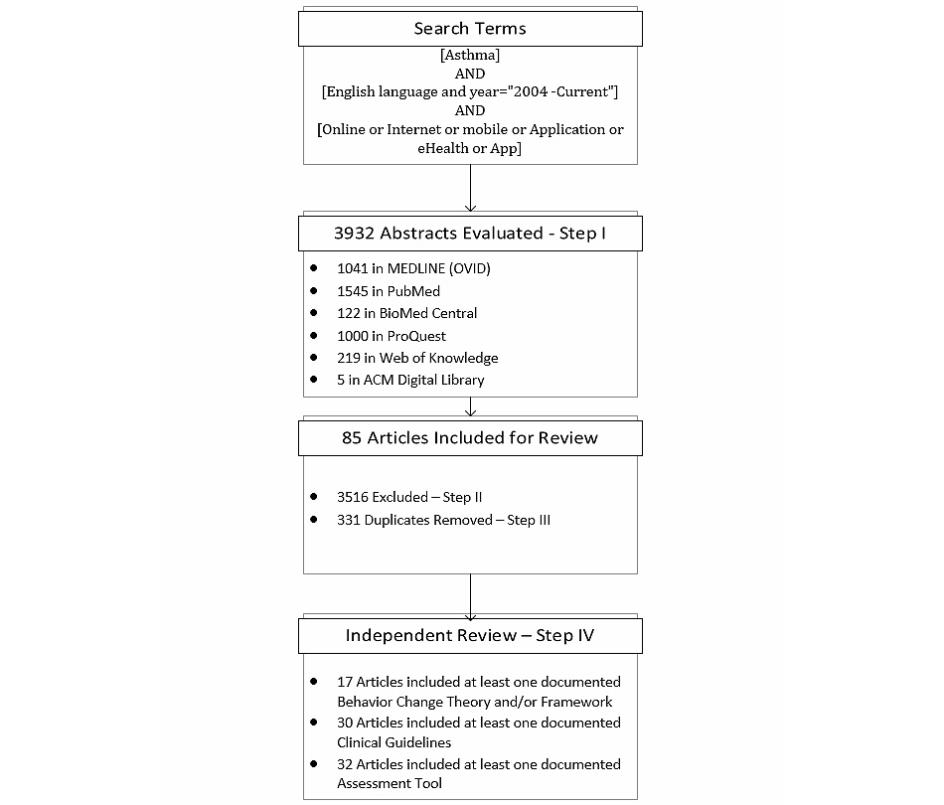
Search results from the six bibliographic databases.

In the last step, the remaining 85 articles were included for independent and in-depth review by the two reviewers. Figure 1 depicts the search breakdown and results for all six bibliographic databases.

The majority of the reviewed studies and interventions reported the following key targeted behaviors [9-13]: (1) managing environmental triggers, (2) accessing asthma services, (3) medication adherence, (4) monitoring peak flow regularly by using portable meters, (5) keeping rescue inhaler accessible, and (6) smoke reduction or cessation.

The findings of the review results will be discussed in three different sections: Theoretical Frameworks, Clinical Guidelines and Assessment Tools, and Other Reviews.

### Theoretical Frameworks

#### Overview

The motive behind conducting this review was to answer the primary research question with respect to whether existing Internet-based interventions for asthma were founded on any behavioral-change theories. And if so, to what extent did these theoretical frameworks inform the design and evaluation of these interventions?

This review identified 17 out of 85 interventions (20%) where at least one model, framework, and/or construct of a behavioral change theory was applied. This implies that the majority of our reviewed interventions did not apply any documented behavioral change theory to inform the design of their interventions. This finding is consistent with what is reported in the literature. Theory-driven strategies for aiding individuals in changing or managing health behaviors are lacking [9].

As such, this review found that there are only a limited number of well-established behavioral change theories and models that were referenced and applied across multiple studies. In total, the reviewers were able to identify 10 behavioral change theories and models that were applied across multiple interventions versus 13 other theories and models that were only applied once within the context of a single study and/or intervention.

Table 1 provides a list of all applied theoretical frameworks and models that were identified across the 85 reviewed interventions.

In the following sections, the theoretical frameworks that were applied in more than three studies will be further analyzed and discussed.

**Table 1:** Applied theoretical frameworks and models of the 85 reviewed Internet-based asthma interventions.

Theoretical frameworks	Number of studies	Cited interventions
Gamification	4	[13-16]
Health Belief Model	4	[10-12,17]
Tailoring	4	[10-13]
Transtheoretical Model	4	[9,10,12,17]
Attribution Theory	3	[11,13,17]
Chronic Care Model	2	[18,19]
Motivational Interviewing	2	[11,17]
Self-Determination Theory	2	[17,20]
Social Cognitive Theory	2	[13,21]
Technology Acceptance Model	2	[22,23]
Biobehavioral Family Model (BFM)	1	[16]
Dual Processing Theory	1	[13]
Ecological Systems Theory	1	[24]
Instructional Theory	1	[13]
Intervention Mapping	1	[13]
Marlatt’s Theory of Relapse	1	[17]
Motivational Theory	1	[13]
Norma Engaging Multimedia Design (NEMD)	1	[16]
Peplau’s Theory of Interpersonal Relations	1	[22]
Social Learning Theory	1	[25]
Sociohistoric Theory	1	[13]
The eHealth Behavior Management Model	1	[9]
Theory of Planned Behavior	1	[9]
Watson’s Model of Caring	1	[22]

#### Gamification

In the past, computer and video games were perceived to be a waste of time and harmful in many aspects to those who play such games excessively, especially for the child and adolescent age groups [26,27]. Nevertheless, the advancement in audio-visual and telecommunication technologies has ignited a new era for today’s games. While the term “gamification” is still evolving, it could be defined as “the use of video game elements in non-gaming systems to improve user experience (UX) and user engagement” [28].

There is a growing body of evidence emphasizing the potential benefits of the social, health, and educational science behind computer games [29]. The potential application of computer games in the health domain was well addressed by the Games for Health projects. The project has defined a taxonomy to depict five main types of games used in health care: Preventative, Therapeutic, Assessment, Educational, and Informatics [27].

This review has shown that principles, concepts, and strategies of gamification were only applied in four studies. However, these four studies only targeted children up to 12 years old [13-16]. The reviewers could not cite any Internet-based asthma interventions employing gamification concepts for the adolescent or adults’ population groups.

In one study conducted in 2013, an online peer support group for asthmatic children used an existing commercial networking website, Club Penguin, to help asthmatic children deal with difficult situations in an engaging manner [14].

Another two studies reported the success of an award-winning program called “Okay With Asthma”, where an interactive digital story was developed and delivered online to support children with their asthma and psychosocial management strategies. This was done through leveraging and employing a novel behavioral model, the Biobehavioral Family Model [15,16].

The “Okay With Asthma” program successfully used the five factors (simulation interactivity, construct interactivity, immediacy, feedback, and goals) identified by the Norma Engaging Multimedia Design model to design its usability and feasibility testing approach [16,30].

Simulation interactivity describes the child’s ability to ‘become’ a character in the story, whereas construct interactivity refers to the availability of activities for the child to create or build in the virtual world. Immediacy is the user’s ability to observe all the actions and interactions that take place in the system. Children need feedback to show that their choices matter; without consequences, there would be no point in performing the actions. The model’s final tenet is goal setting. Whether the goal is set extrinsically (by the game developer) or intrinsically (the child determining own goals), it is important for there to be goals to achieve. [31]

As well, the Watch-Discover-Think-Act (WDTA) study [13] provided an applied example of how behavioral and motivation theories could be translated within the context of gamification. The WDTA program developed a game that walks through 18 real-life and four fantasy scenarios. The players, who are children with asthma, have to complete a set of tasks related to asthma self-management in order to progress across scenarios. Feedback is provided as a reinforcement of information for the children and their parents.

As depicted in Table 2, the reviewers were able to validate the translation steps of the behavioral change theoretical methods in the “Watch-Discover-Think-Act” [13] study against a number of other studies, such as the five factors of the Norma Engaging Multimedia Design model that were applied within the context of “Okay With Asthma” [16]. The correlation between the findings of those two different studies validates the impact and influence gamification theories and methods could have to increase patients’ motivation, self-efficacy, and engagement level within the context of Internet-based asthma interventions.

**Table 2:** Correlation between the applied theoretical methods and factors of the two studies, “Watch-Discover-Think-Act” and “Okay With Asthma”.

WDTA (Watch-Discover-Think-Act)	Okay With Asthma
Personalized Information	Simulation Interactivity
Fantasy Context + Multiple Modalities	Construct Interactivity
Learner Control	Immediacy
Reinforcement	Feedback
Goal Settings	Goals

#### Health Belief Model

The Health Belief Model dates back to the 1950s, initially developed by Hochbaum (1958) and Rosenstock (1960), and then extended by Kirscht and Becker in 1974 [32]. The Health Belief Model is a theoretical framework that attempts to study and predict the individual’s health preferences and actions based on observed attitudes and personal beliefs. The model explains the individual’s motivation to take a health care-related action based on the following factors:

(1) The existence of sufficient motivation (or health concern) to make health issues salient of relevant.

(2) The belief that one is susceptible (vulnerable) to a serious health problem or to the sequelae of that illness or condition. This is often termed perceived threat.

(3) The belief that following a particular health recommendation would be beneficial in reducing the perceived threat, and at a subjectively-acceptable cost. [33]

The Health Belief Model was applied in the context of providing individualized messages and communication with patients to promote self-efficacy and better patient engagement [10-12,17].

The Puff City program that was evaluated in six Detroit high schools and reported in four different studies [10-12,17] identified and evaluated three core behaviors: namely, controller medication adherence, rescue inhaler availability, and smoking cessation/reduction. In the event of a negative change in any of the three core behaviors, theory-based health messages and information on asthma control were sent to the patients to sustain their self-efficacy and asthma self-regulation [12].

#### Tailoring

In the literature, “tailoring” is defined as “…assessment and provision of feedback based on information that is known or hypothesized to be most relevant for each individual participant of a program” [11,34,35], and “…any combination of information or change strategies intended to reach one specific person, based on characteristics that are unique to that person, related to the outcome of interest, and have been derived from an individual assessment” [10,35].

A number of studies and interventions have pointed out the significance of identifying the resistant groups at earlier stages of the intervention. These groups are less motivated to change their behavior and take ownership in managing their asthma. The objective is to use “tailoring” as a means to apply behavioral change theories, such as the Transtheoretical Model and the Health Belief Model, to motivate those subgroups and achieve positive changes in their behaviors with respect to their self-efficacy and asthma management [10-13]. The reviewed studies have utilized “tailoring” to customize the communication and education strategies with the targeted resistant subgroup based on their beliefs, attitude, and personalized information.

#### Transtheoretical Model

The Transtheoretical Model promotes individuals to change their behaviors for a healthier lifestyle.

The Transtheoretical Model is based on the premise that individuals are in one of five possible stages of change associated with a particular behavior. Precontemplation is the stage in which a person has no interest in changing the behavior. Contemplation is when a person would like to change the behavior someday but is not yet ready. Preparation is when a person is ready to make the change but needs assistance in moving that want into reality. The more active stages include Action and Maintenance. Those in Action have begun the behavior change process. Key to their success is moving the change to Maintenance, where change takes place over time. [9,36]

The review identified three different studies where the concepts of the Transtheoretical Model were applied in the methods’ design and patients’ assessment through the stages of the change [9,10,12,17].

The Asthma Management Demonstration Project was developed to manage the following four asthma-related behaviors for asthma patients among employees and students of Western Michigan University: monitoring peak flow measurements, accessing asthma services, using prescription asthma medications properly, and managing environmental triggers [9]. Based on concepts of Transtheoretical Model, the project developed transactional questioning to stage their asthma patients according to their readiness to change their asthma-related behavior [9].

The Web-based Puff City program has also applied concepts of Transtheoretical Model to motivate their patients to change three core asthma-related behaviors: controller medication adherence, rescue inhaler availability, and smoking cessation/reduction [10,12,17].

### Clinical Guidelines and Assessment Tools

#### Overview

In response to our secondary research question, we found that the application and employment of clinical guidelines and assessment tools were more abundant than theoretical frameworks across the reviewed interventions. The review identified 30 out of 85 interventions (35%) where at least one documented clinical guideline was applied. In total, there were six clinical guidelines applied across the 30 identified interventions as listed in Table 3.

As such, the review identified 32 out of 85 interventions (38%) where at least one documented assessment tool was applied. In total, there were 21 assessment tools applied across the 32 identified interventions as listed in Table 4.

This review found that many guidelines and assessment tools were broadly adopted by a relatively large number of interventions, for example, the National Asthma Education and Prevention Program referenced across 15 (of the 85) interventions [10-12,16-18,20-22,37-42], the Global Initiative for Asthma (GINA) guidelines referenced across 13 interventions [43-55], and the Asthma Control Questionnaire referenced across 11 interventions [19,20,44,49,56-62].

**Table 3:** Applied clinical guidelines of the 85 reviewed Internet-based asthma interventions.

Clinical guidelines	Number of studies	Cited interventions
National Asthma Education and Prevention Program (NAEPP)	15	[10-12,16-18,20-22,37-42]
Global Initiative for Asthma (GINA) Guidelines	13	[43-55]
British Guideline on the Management of Asthma	1	[54]
Canadian Asthma Consensus Guidelines (CACG)	1	[63]
International ERS/ATS Guidelines on Definition, Evaluation and Treatment of Severe Asthma	1	[54]
Standards for the Diagnosis and Treatment of Patients with COPD	1	[47]

**Table 4:** Applied assessment tools of the 85 reviewed Internet-based asthma interventions.

Assessment tools	Number of studies	Cited interventions
Asthma Control Questionnaire (ACQ)	11	[19,20,44,49,56-62]
Asthma Quality of Life Questionnaires (AQLQ)	9	[19,44,48,50,59,62,64-66]
Pediatric Asthma Quality of Life Questionnaire (PAQLQ)	8	[43,50,52,53,57,61,67,68]
Asthma Control Test (ACT)	8	[42,51-53,64,67-69]
International Survey of Asthma and Allergies in Childhood (ISAAC) questionnaire	4	[11,12,55,68]
Asthma Therapy Assessment Questionnaire (ATAQ)	3	[49,57,60]
Child Asthma Control Test (C-ACT)	3	[52,53,68]
Mini Asthma Quality of Life (Mini AQLQ)	3	[56,58,63]
Knowledge, Attitude and Self-Efficacy Asthma Questionnaire (KASE-AQ)	2	[58,60]
Pediatric Asthma Caregivers Quality of Life Questionnaire (PACQLQ)	2	[52,53]
The Asthma Life Quality Questionnaire (ALQ)	2	[56,64]
The Consumer Asthma Knowledge Questionnaire and inhalation technique with the standardized checklist of the Dutch Asthma Foundation	2	[57,59]
Air Quality Health Index (AQHI)	1	[63]
Allergic Rhinitis and its Impact on Asthma Questionnaire (ARIA)	1	[68]
Asthma Behavior Checklist (ABC)	1	[25]
Asthma Self-Regulatory Development Interview	1	[17]
Children’s Health Survey for Asthma (CHSA) by the American Academy of Pediatrics	1	[70]
Eyberg Child Behavior Inventory (ECBI)	1	[25]
Illness Management Survey (IMS)	1	[67]
Multidimensional Scale of Perceived Social Support	1	[11]
The Royal College of Physicians’ “Three Key Questions”	1	[71]

#### International Asthma Guidelines

With the aim to employ an evidence base to reduce asthma prevalence, morbidity, and mortality, the Global Initiative for Asthma (GINA) was launched in 1993 as a collaboration between the National Heart, Lung, and Blood Institute, the National Institutes of Health, and the World Health Organization [72]. The GINA guidelines were referenced in 13 of the 85 reviewed interventions [43-55].

Another example of internationally applied asthma guidelines is the International Survey of Asthma and Allergies in Childhood (ISAAC) questionnaire. Established in 1991, the ISAAC guidelines aimed to investigate asthma in the pediatric population as a measure to control the increasing conditions on the global scale [73]. Items from the ISAAC guidelines were included in the Lung Health Survey in four of the reviewed interventions [11,12,55,68].

#### National Asthma Guidelines

In response to the increasing asthma challenges in the United States, the National Asthma Education and Prevention Program (NAEPP) was initiated in March 1989 by the National Heart, Lung, and Blood Institute (NHLBI) [74]. This review has shown that the NAEPP guidelines were the most referenced asthma guidelines across all reviewed interventions as they were implemented in the design of 15 out of the 85 reviewed interventions [10-12,16-18,20-22,37-42].

This review identified a number of national clinical guidelines that were adopted and applied in a smaller number of interventions conducted at the national level, such as the British Guideline on the Management of Asthma [54], the Canadian Asthma Consensus Guidelines (CACG) [63], and the Air Quality Health Index (AQHI) in Canada, as well as the standardized checklist of the Dutch Asthma Foundation [57,59].

#### Pediatric Asthma Guidelines

In addition to the national and international guidelines, the review also identified a number of children-specific guidelines such as the Pediatric Asthma Quality of Life Questionnaire (PAQLQ) [43,50,52,53,57,61,67,68], International Survey of Asthma and Allergies in Childhood (ISAAC) questionnaire [11,12,55,68], the Eyberg Child Behavior Inventory (ECBI) [25], the Children’s Health Survey for Asthma (CHSA) by the American Academy of Pediatrics [70], and the Child Asthma Control Test (C-ACT) [52,53,68].

### Other Reviews

A total of 14 other reviews of Internet- and electronic-based asthma interventions were identified. These reviews did not evaluate Internet-based asthma interventions with respect to the evidence base around the behavioral change theoretical frameworks, applied clinical guidelines, and assessment tools. However, the identified other reviews share similar discussions around main topics such as patients’ perception of Internet-based interventions, limitation of existing studies, and the effect of evolving Internet and mobile technologies on the relationship between asthma patients and their health care providers.

Six of the 14 reviews indicated that Internet-based interventions were well-perceived by asthma patients and their usage was associated with promoting positive health behaviors among asthma patient groups [6,7,75-78]. On the other hand, a number of reviews reported that numerous studies for existing interventions were conducted on a small group of subjects for a limited, and often short, period of time resulting in mixed results with respect to controlling asthma symptoms and improving quality of life for asthma patients [1,7,79-81]. Last, four reviews shared concerns pertaining to the increased diffusion of Internet and mobile technologies into the delivery of care and to its impact on the clinician-patient relationship that could have negative effect on both patients and health care providers [1,7,79,80].

##  Discussion

### Principal Findings

In an attempt to answer the primary research question pertaining to the evidence base around the behavioral change, self-care, and self-management theoretical frameworks applied within the context of the reviewed Internet-based asthma interventions, the reviewers identified 17 out of 85 interventions (20%) where at least one model, framework, and/or construct of a behavioral change theory was applied. This implies that the majority of our reviewed interventions did not apply any documented behavioral change theory to inform their design. As such, this review found that only a limited number of behavioral change theories and models were referenced and applied across multiple studies.

In total, the reviewers were able to identify 10 behavioral change theories and models that were applied across multiple (more than one) interventions versus 13 other theories and models that were only applied within the context of a single study and/or intervention.

Compared to the applied theoretical frameworks, and in response to the secondary research question, the reviewers were able to report that the application and employment of clinical guidelines and assessment tools were more salient across the reviewed interventions. The review identified six clinical guidelines that were applied across 30 of the 85 interventions (35%) as well as a total of 21 assessment tools that were applied across 32 of the 85 interventions (38%).

The National Asthma Education and Prevention Program (NAEPP) guidelines were the most referenced asthma guidelines across all the reviewed interventions as they were implemented in the design of 15 out of the 85 reviewed interventions [10-12,16-18,20-22,37-42].

### Limitations

This review has a number of limitations. First, the reviewers searched the literature in six major bibliographic databases between 2004 and October 2014 only; there may be other relative studies published in other databases. Second, the reviewers did not employ any theory or guidelines to evaluate the quality of each included and reviewed study. Thus, all reviewed studies are assumed to be of the same quality.

### Conclusions

It was found that the majority of published interventions did not apply behavioral change theory, clinical guidelines, and/or assessment tools to inform their design. Further, it was found that the application of clinical guidelines and assessment tools were more salient across the reviewed interventions. A consequence, therefore, is that many Internet-based asthma interventions are designed in an ad hoc manner, without the use of any notable evidence-based theoretical frameworks, clinical guidelines, and/or assessment tools.

## Abbreviations

ABC: Asthma Behavior Checklist
ACQ: Asthma Control Questionnaire
ACT: Asthma Control Test
ALQ: Asthma Life Quality Questionnaire
AQHI: Air Quality Health Index
AQLQ: Asthma Quality of Life Questionnaires
ATAQ: Asthma Therapy Assessment Questionnaire
CACG: Canadian Asthma Consensus Guidelines
C-ACT: Child Asthma Control Test
CHSA: Children’s Health Survey for Asthma
COPD: chronic obstructive pulmonary disease
ECBI: Eyberg Child Behavior Inventory
eHealth: electronic health
EHR: electronic health records
EMR: electronic medical records
GINA: Global Initiative for Asthma guidelines
HIS: hospital information system
IMS: Illness Management Survey
ISAAC: International Survey of Asthma and Allergies in Childhood questionnaire
KASE-AQ: Knowledge, Attitude and Self-Efficacy Asthma Questionnaire
Mini AQLQ: Mini Asthma Quality of Life
NAEPP: National Asthma Education and Prevention Program
NHLBI EP3: The National Heart, Lung, and Blood Institute, Expert Panel Report 3
NHLBI: National Heart, Lung, and Blood Institute
PAQLQ: Pediatric Asthma Quality of Life Questionnaire
SMS: short message service
UX: user experience
WDTA: Watch-Discover-Think-Act
WHO: World Health Organization
